# Effects of dietary milk- and soya-phospholipids on lipid-parameters and other risk indicators for cardiovascular diseases in overweight or obese men – two double-blind, randomised, controlled, clinical trials

**DOI:** 10.1017/jns.2016.9

**Published:** 2016-05-20

**Authors:** Anne Weiland, Achim Bub, Stephan W. Barth, Juergen Schrezenmeir, Maria Pfeuffer

**Affiliations:** Department of Physiology and Biochemistry of Nutrition, Max Rubner-Institut, Haid-und-Neu-Strasse 9, D-76131 Karlsruhe, Germany

**Keywords:** Milk phospholipids, Soya phospholipids, Plasma lipids, Human nutrition, CVD, ALT, alanine transaminase, AST, aspartate transaminase, CRP, C-reactive protein, GGT, γ-glutamyl transferase, HDL-C, HDL-cholesterol, HOMA-IR, homeostasis model assessment of insulin resistance, LDL-C, LDL-cholesterol, MFGM, milk fat globule membrane, MTHFR, methylenetetrahydrofolate reductase, PC, phosphatidylcholine, PL, phospholipid, SM, sphingomyelin, TC, total cholesterol, tHcy, total homocysteine

## Abstract

The present study examined the effect of milk phospholipids (milk-PL) on lipid metabolism and on other risk factors for CVD, in comparison with milk fat (control) or soya phospholipids (soya-PL), respectively. Two double-blind parallel-group intervention trials were conducted in overweight or obese male subjects. In the first trial (trial 1), sixty-two men consumed milk enriched with either 2 g milk-PL or 2 g milk fat (control) for 8 weeks. In trial 2, fifty-seven men consumed milk enriched with either 3 g milk-PL or 2·8 g soya-PL for 7 weeks. In trial 1, milk-PL as compared with control reduced waist circumference but did not affect plasma lipids (total, HDL- and LDL-cholesterol, total cholesterol:HDL-cholesterol ratio, TAG, phospholipids), apoB, apoA1, glucose, insulin, insulin sensitivity index, C-reactive protein, IL-6, soluble intracellular adhesion molecule and total homocysteine (tHcy). Serum activities of alanine transaminase and aspartate transaminase were not changed. Activity of γ-glutamyl transferase (GGT), a marker of fatty liver, increased in the control but not in the milk-PL group, with a significant intervention effect. In trial 2, milk-PL as compared with soya-PL did not affect the above-mentioned parameters, but decreased GGT. Subjects with the methylenetetrahydrofolate reductase mutations CT and TT had 11 % (*P* < 0·05) higher baseline tHcy concentrations than those with the wild-type CC. However, genotype did not modulate the phospholipid intervention effect on tHcy. In conclusion, supplementation with milk-PL as compared with control fat reduced waist circumference and, as compared with both control fat and soya-PL, GGT activity.

A high cholesterol concentration, particularly in LDL, and a low HDL-cholesterol (HDL-C) concentration are accepted risk factors for CVD. Obesity, particularly abdominal obesity, is an independent risk factor for CHD in middle-aged men^(^^1^^,^^2^^)^. Obesity promotes alterations in other intermediate risks such as dyslipidaemia, glucose intolerance and inflammatory state^(^^2^^)^. Non-alcoholic fatty liver disease promoted by obesity is also associated with an increased prevalence of CVD^(^^3^^,^^4^^)^.

Phospholipids (PL) belong to the dietary components which may affect cholesterol metabolism. They are polar lipids which occur in all animal and plant membrane structures. The human diet provides 2–8 g PL per d, which represents 1–10 % of total fat intake. Foods with a high PL content include eggs, meats, fish, grains and oilseeds. The most common PL in foods is phosphatidylcholine (PC). Sphingomyelin (SM), a P-containing sphingolipid, is solely present in animal food sources, i.e. in eggs, meat and fish, and is ingested at a level of 0·3–0·4 g/d^(^^5^^)^.

Previously mostly soya-PL (trivial name soyabean lecithin) were tested in the course of animal and human feeding trials. They are rich in PUFA, mainly linoleic acid. In animal studies soya-PL inhibited cholesterol absorption, decreased plasma cholesterol and also liver lipids, and protected against the development of atherosclerosis (for a review, see Cohn *et al*.^(^^5^^)^). Small-size short-term human studies showed that soya-PL may increase HDL-C^(^^6^^)^ and apoA1^(^^6^^,^^7^^)^.

Animal polar lipids differ from soya-PL in several aspects. Only animal PL contain SM. Particularly their PC and SM classes are rich in SFA. The majority of milk polar lipids, mostly PL (milk-PL), are contained in the milk fat globule membrane (MFGM)^(^^8^^)^ and are closely associated with proteins^(^^9^^)^. In fact, animal-source PL or ‘saturated’ PL showed high bioactivity. Milk-SM inhibited cholesterol absorption in mice more than egg-PC, despite a similar fatty acid pattern^(^^10^^)^. Milk-PL at a dose of 0·5 or 1·2 % decreased plasma cholesterol in mice on a high-fat diet^(^^11^^,^^12^^)^ but not on a regular-fat chow diet^(^^11^^)^. Recently published well-designed human studies found no effect of milk-PL on plasma lipids^(^^13^^,^^14^^)^, but a significant effect of PL-rich buttermilk^(^^15^^)^, a favourable trend of buttermilk combined with egg yolk as compared with egg alone^(^^16^^)^, and of milk fat with intact MFGM as compared with pure milk fat^(^^17^^)^.

Metabolic effects of PL go beyond those on cholesterol absorption and plasma lipids (for a review, see Kullenberg *et al*.^(^^18^^)^). In mouse or rat experimental models, soya-PL^(^^19^^)^, as well as milk-PL^(^^11^^,^^12^^,^^20^^)^ and SM^(^^21^^)^ decreased liver lipids. An SM-supplemented diet increased hepatic gene expression of PPARα, and decreased expression of stearoyl-CoA desaturase-1 (SCD1) in Zucker fatty rats^(^^21^^)^. Also, mice on a high-fat milk-PL-enriched diet showed reduced hepatic expression of SCD1 and fatty acid synthase genes^(^^11^^)^. Lower SCD1 activity is linked to improved liver lipid metabolism and insulin sensitivity^(^^22^^)^.

PC and SM are important dietary sources of choline. The European Food Safety Authority has approved claims for dietary choline with respect to lipid metabolism, liver function and homocysteine (Hcy) metabolism^(^^23^^)^. The Institute of Medicine considers 550 mg/d choline as adequate intake for men^(^^24^^)^. Most subjects on an experimental choline-deficient diet developed fatty liver, experienced muscle damage, or both, within a few weeks^(^^25^^)^. Choline metabolism, methionine metabolism and folate metabolism are interrelated. The dietary requirement for choline varies substantially among individuals, and is modulated by SNP in genes of choline and folate metabolism^(^^26^^)^, including methylenetetrahydrofolate reductase (MTHFR)^(^^27^^,^^28^^)^.

Based on the hypothesis that PL are relevant for the beneficial health effects of dairy products^(^^29^^)^, we studied the metabolic effects of milk-PL. The first study assessed the metabolic effects of milk-PL as compared with the same amount and pattern of fatty acids provided as milk fat. In a second step the effects of milk-PL were compared with those of soya-PL, i.e. PL with both a different PL class pattern and a different fatty acid pattern. The main focus of these studies was on the treatment effects on the total cholesterol (TC):HDL-C ratio. The TC:HDL-C ratio allows the best prediction of future coronary events, closely followed by the apoB:apoA1 ratio^(^^30^^)^. In order to cover further parameters relevant to CVD risk, plasma parameters of lipid and carbohydrate metabolism, various inflammation markers, total Hcy (tHcy) and liver enzyme activities were measured. Furthermore, the polymorphism MTHFR 677, which may affect choline and betaine metabolism, respectively, was determined, as a potential genetic determinant affecting PL bioactivity.

## Subjects and methods

### Subjects

Participants were recruited through the Max Rubner-Institut database and local advertisements in Karlsruhe and the surrounding area, Germany. Men were eligible if between 50 and 76 years of age, overweight (BMI ≥ 27 kg/m^2^) and non-smokers. Exclusion criteria for both trials were the use of drugs affecting lipid metabolism, acute or chronic gastrointestinal diseases (e.g. diseases affecting nutrient absorption, digestion, metabolism or excretion), diabetes (fasting glucose ≥7·0 mmol/l), milk protein allergy or lactose intolerance, and furthermore soya protein allergy in trial 2. The studies were conducted according to the guidelines laid down in the Declaration of Helsinki and all procedures involving human subjects were approved by the ethics committee of the State Medical Chamber of Baden-Württemberg (F 2010-020 and F 2010-089). Written informed consent was obtained from all subjects prior to the studies. The studies were registered at https://drks-neu.uniklinik-freiburg.de/drks_web as DRKS00000383 (trial 1) and DRKS00003125 (trial 2). Participants were asked to consume no more than one alcoholic drink per d, and not to change their eating habits and physical activity routine during the trials. The use of supplemental vitamins, minerals, phytochemicals or *n*-3 oil supplements was forbidden. A list of products which should be avoided was provided. The subjects were requested to inform the study physician about any newly prescribed medications.

### Test materials

Milk-PL preparations (Lipamin M20; Lecico) were isolated from butter serum. PL were quantified by ^31^P-NMR-spectroscopy (Spectral Service). The Lipamin M20 lot used for trial 1 contained 18·4 % PL, 31·6 % total fat, 51·6 % protein and 5·4 % lactose. The lot used for trial 2 contained 31·2 % PL, 41·2 % total fat, 46·0 % protein and 1·2 % lactose. PC and SM represented 29·2 and 23·1 % of total PL in the lot for trial 1 and 29·5 and 25·1 % in the lot for trial 2. The soya-PL preparation (deoiled soya lecithin P 900 IPM; Lecico) used for trial 2 contained 91 % total fat and 72 % PL according to the manufacturer, but only 66·1 % PL as later determined by ^31^P-NMR-spectroscopy. PC represented 34·7 % of the total soya-PL.

### Study design trial 1

This trial was performed in spring 2010 in a randomised, double-blind and parallel design. Eligible participants were randomly assigned following simple randomisation procedures (computerised random numbers between 0 and 1) to one of two treatment groups. In all, seventy-six men were screened for participation; sixty-three were included in the study. One dropped out when he started to use non-permitted medication, so sixty-two men completed the trial. During the 8-week intervention the participants consumed daily 200 ml of PL-enriched drink (containing 2 g milk-PL) or 200 ml of control drink (containing 2 g milk fat). The PL-drink was semi-skimmed milk (1·5 % fat) supplemented with 5·4 % Lipamin M20 powder (Lecico). To make up for protein, lactose and lipids of the Lipamin M20 powder, the control drink was prepared by enriching semi-skimmed milk with cream and skimmed milk concentrate. Composition of both test drinks is described in Table 1. They were manufactured and bottled in enhanced shelf-life quality packaging (Unternehmensgruppe Theo Müller). Fresh batches were provided and picked up by the participants every 2 weeks. Drinks were stored refrigerated until consumption. Appearance, taste and packaging of the drinks were identical and bottles (200 ml volume) were coded in such a way that participants and staff were unaware of the drink allocation. Participants were asked to consume the drinks with a meal.

**Table 1.** Composition of the test drinks*

	Trial 1		Trial 2	
	Control	Milk-PL	Soya-PL	Milk-PL
Milk (1·5 % fat) supplemented with…	Cream 4·7 %, SMC (10·6 %)†	Lipamin M20 (5·4 %)‡	P 900 IBM (1·67 %)	Lipamin M20 (3·85 %)‡
Chemical composition				
Fat (%)	3·4	3·2	2·5	2·6
Protein (%)	4·3	5·7	3·4	5·0
Lactose (%)	6·2	4·8	4·4	4·5
DM (%)	14·6	14·9	11·7	13·7
Ca (%)	0·186	0·173	0·123	0·137
Absolute amounts provided per d with the test drinks				
Volume (ml)	200	200	250	250
Fat (g)	6·8	6·4	6·3	6·4
PL (g)§	0·0	2·0	2·8	3·0
Choline (mg)§‖	0	164	156	264
Energy (kJ)¶	623	604	577	659

PL, phospholipid; SMC, skimmed milk concentrate.

* Chemical analysis was done after blending the carrier milk with supplements. For trial 1, values are the mean of four batches, each determined in triplicate.

† Cream with 39 % fat and SMC with 35 % DM, 0·26 % fat.

‡ Lipamin M20 lot 1 and lot 2, respectively.

§ Values for supplements only. Concentrations in the milk used for the test drinks (approximately 0·029 g/100 g PL and 16·1 mg/100 g choline) not included.

‖ Choline supply is calculated from phosphatidylcholine and sphingomyelin concentrations in the supplements.

¶ Calculated value.

### Study design trial 2

This trial was performed in spring 2011. Of the seventy-one men screened for participation, fifty-eight were included in the study. One man in the milk-PL group dropped out because of suspected allergic reactions, so fifty-seven men completed the trial. Thirty-eight of these men also had participated in trial 1. During the 7-week intervention the participants consumed daily 250 ml of test drink that provided either 3 g milk-PL or 2·8 g soya-PL. The drink was semi-skimmed milk (1·5 % fat) supplemented with 3·85 % Lipamin M20 powder or 1·67 % soya lecithin (P 900 IBM) (both Lecico). Calculation of the soya-PL supplemental dose was based on the manufacturers’ data, to yield 3 g per 250 ml. However, based on the ^31^P-NMR-spectroscopy data, the final PL concentration was somewhat lower, namely 2·8 g per 250 ml. Milk drinks for this study were manufactured and bottled in ultra-high temperature treatment (UHT) quality packaging (Milchwerke Mittelelbe) and handed out to the participants at the start of the trial. Drinks were identical in appearance and taste. Bottles (250 ml volume) were coded. Again, participants were asked to consume the drinks with a meal.

### Diet protocols

Participants completed 4-d food protocols (according to Koebnick *et al*.^(^^31^^)^, with minor modifications) during and outside the intervention periods. Nutrient intake analysis was done using PRODI 5.7 software and Bundeslebensmittelschlüssel (BLS) version II.3 (trial 1), or Prodi® version 5.9 and BLS version 3.0 (trial 2). Calculation of PL intake was based on recorded food items and PL concentrations reported in the literature. As there were only few data available, the calculated values can give only a rough approximation of the true intake. The USDA nutrient database 2004 for choline (https://www.ars.usda.gov/SP2UserFiles/Place/80400525/Data/Choline/Choln02.pdf) was used to estimate choline intake.

### Blood sampling

Fasting venous blood samples were collected before starting the intervention (day zero) and after the intervention, between 07.30 and 10.00 hours. EDTA whole blood (for glutathione determination and DNA preparation), plasma (EDTA, lithium heparin) and serum samples were obtained. When required, samples were stored frozen at −80°C until analysis.

### Anthropometric and blood pressure measurements

Anthropometric measures including waist and hip circumferences and blood pressure measurements were taken at the beginning and at the end of the intervention periods and recorded following standard operation procedures according to WHO 2008^(^^32^^)^. BMI was calculated by dividing body mass (in kg) by height squared (in m). Bioelectrical impedance analysis (SensorMedics) was used to determine percentage of body fat.

### Analytical methods

Fasting plasma or serum samples were analysed for routine clinical parameters as well as concentrations of TAG, TC, HDL-C and LDL-cholesterol (LDL-C), glucose, and γ-glutamyl transferase (GGT) and aspartate transaminase (AST) activities on the same day by an accredited routine laboratory (MVZ Labor Seelig). ApoA1, apoB and alanine transaminase (ALT) activity were assessed enzymatically using the Konelab 20i clinical analyser (Thermo Scientific). Total glutathione in whole blood was measured according to Becker *et al*.^(^^33^^)^. Serum concentrations of insulin and high-sensitivity C-reactive protein (CRP) were measured by ELISA (DRG Instruments) as well as soluble intracellular adhesion molecule and IL-6 (R&D Systems). Serum PL were analysed by an enzymic assay (WAKO Chemicals). Plasma tHcy concentrations were assessed by HPLC with fluorimetric detection according to Toyo'oka & Imai^(^^34^^)^. Homeostasis model assessment of insulin resistance (HOMA-IR) was calculated as ((fasting insulin mU/l × fasting glucose mmol/l)/22·5) according to Matthews *et al*.^(^^35^^)^. For determination of the MTHFR 677 genotype (rs1801133), peripheral blood mononuclear cell genomic DNA was prepared using a microspin kit (NucleoSpin blood; Macherey-Nagel) and further processed utilising a PCR-based high-resolution melting curve analysis method carried out on a LightCycler® 480 instrument with subsequent data processing by gene scanning software (Roche). The respective PCR primer (5′→3′) sequences were: (forward) >>ccc aaa ggc cac ccc gaa gc<< and >>gcc cat gtc ggt gca tgc ct<< and the according PCR protocol consisted of thirty-nine cycles of denaturation, annealing and extension (95°C, 10 s; 60°C, 15 s; 72°C, 1 s) and a subsequent melting analysis.

### Statistical analysis

A previous study providing 2·8 or 5·8 g/d PL^(^^6^^)^ suggested that a PL-induced 6 % decrease in the TC:HDL-C ratio is realistic. Based on another study showing a 0·29 mmol/l (6·3 %) decrease in the TC:HDL-C ratio along with a standard deviation of 0·45 mmol/l in response to dietary modification, and with a baseline TC:HDL-C ratio of 4·5^(^^36^^)^, similar to that in our participants, the required total number of participants completing the study was calculated as *n* 58 (α = 0·05 two-tailed, power = 90 %). Data are expressed as mean and standard deviation. Data were tested for normality (Shapiro–Wilk) and variance homogeneity (Levene). Intervention effects were analysed by repeated-measures ANOVA if datasets were normally distributed and showed variance homogeneity. For datasets with asymmetric distribution delta concentrations were compared by *t* test or Mann–Whitney rank-sum test. Changes of tHcy concentration as a function of MTHFR 677 genotype (CC *v*. CT and TT combined) were assessed by the Kruskal–Wallis *H* test. Analyses were carried out using SPSS Inc. PASW 17 software. The level of significance was set at *P* < 0·05.

## Results

### Baseline characteristics

Baseline characteristics of the participants who completed trials 1 and 2 are presented in Table 2. Participants were either overweight (40·3 % in trial 1 and 56·1 % in trial 2) or obese (59·7 % in trial 1 and 43·9 % in trial 2) and all had increased waist circumference, body fat mass and TC concentration. Baseline parameters were not significantly different between the two groups within each trial, and similar between trials 1 and 2 (Table 2). The distribution of the MTHFR 677 genotype in trial 2 was CC (*n* 23; 40·4 %), CT (*n* 32; 56·1 %) and TT (*n* 2; 3·5 %).

**Table 2.** Baseline characteristics of the participants (Mean values and standard deviations)

	Trial 1 (*n* 62)		Trial 2 (*n* 57)	
Variable	Mean	sd	Mean	sd
Age (years)	62·5	6·4	63·1	6·5
Weight (kg)	94·5	10·2	93·3	9·7
BMI (kg/m^2^)	30·8	2·4	30·4	2·4
Waist circumference (cm)	109·6	7·2	108·2	6·4
Body fat mass (%)	31·0	2·5	31·1	2·9
Total cholesterol (mmol/l)	5·87	0·89	5·60	0·88
HDL-cholesterol (mmol/l)	1·37	0·34	1·31	0·30
TAG (mmol/l)	1·44	0·66	1·49	0·55
Glucose (mmol/l)	5·50	0·57	5·44	0·54
ApoA1 (g/l)	1·60	0·23	1·62	0·21
ApoB (g/l)	1·27	0·25	1·37	0·21

### Dietary intake and compliance

Energy intake and nutrient composition of the habitual diets during the intervention periods of trials 1 and 2 are given in Table 3. Carbohydrate and energy intakes was slightly higher in the soya-PL as compared with the milk-PL group in trial 2 (*P* < 0·05). Otherwise there were no differences between intervention groups, and there were no significant differences in energy and nutrient intakes during the intervention and outside the intervention period in both studies. Nutrient intake was comparable with that of the German average population of that age group as assessed in the Nationale Verzehrstudie II (2008) (https://www.bmel.de/SharedDocs/Downloads/Ernaehrung/NVS_ErgebnisberichtTeil2.pdf?__blob=publicationFile). Major sources of PL in foods were meat and meat products (41·9 and 41·7 %, respectively) and egg and egg products (30·3 and 29·2 %, respectively). Choline intake was similar to that of US populations^(^^37^^)^. Major sources of choline were also meat and meat products (34·3 and 33·0 %, respectively) and egg and egg products (18·5 and 17·9 %, respectively), closely followed by the food group bread, pastries and grain products (16·6 and 16·4 %, respectively).

**Table 3.** Energy and nutrient intakes during the intervention periods* (Mean values and standard deviations of 4-d protocols)

	Trial 1 (*n* 62)		Trial 2 (*n* 57)	
	Mean	sd	Mean	sd
Energy (MJ/d)	9·49	2·18	9·57†	1·99
Protein (% energy)	17·9	4·0	17·4	4·3
Fat (% energy)	36·1	11·7	34·9 8	4·8
Carbohydrates (% energy)	44·1	11·3	45·8†	9·8
Mono-/disaccharides (% energy)	19·6	7·6	20·1	7·6
Dietary fibre (g/d)	27·1	8·1	25·4	7·8
Cholesterol (mg/d)	421	152	444	227
Ca (mg/d)	991	332	924	270
Vitamin B_6_ (mg/d)	1·9	0·5	1·9	0·5
Folate (μg/d)	280	86	355	151
Vitamin B_12_ (μg/d)	7·7	4·3	8·2	7·5
Phospholipids (g/d)	3·08	1·28	3·00	1·75
Choline (mg/d)	384	117	387	156

* All foods including carrier milk, excluding supplements.

† Energy and carbohydrate intakes were 9·7 and 12 % higher in the soya-phospholipid group as compared with the milk-phospholipid group in trial 2 (*P* = 0·049 and 0·035, respectively).

Participants consumed the test drinks in addition to the normal diet. Test drinks were well tolerated. Subjects did not report side effects, except for one person in trial 2. There were no objective chemical measures (blood parameters) available to assess compliance. But as participants kept in regular contact with the study centre, and on the basis of the dietary records provided, appropriate compliance was assumed.

### Intervention effects trial 1

Milk-PL as compared with control did not change the lipid parameters, namely TC, HDL-C, LDL-C, TAG, PL, the primary parameter TC:HDL-C ratio as well as apoA1 and apoB. Likewise, concentrations of glucose, insulin and HOMA-IR did not change (Table 4). Milk-PL as compared with the control treatment did not change concentrations of CRP, IL-6, soluble intracellular adhesion molecule as well as tHcy (Table 4). Due to technical problems, valid glutathione data were only available at the end of the intervention. Values did not differ between groups (0·81 (sd 0·29) *v*. 0·80 (sd 0·24) mmol/l whole blood) for the control and milk-PL groups, respectively. Concerning liver enzyme activities, there were no significant intervention effects on ALT and AST. There was a significant 16 % increase of GGT activity in the control group (*P* < 0·001), but no change in the milk-PL group, and thus a significant intervention effect (*P* < 0·001) (Table 4). However, enzyme activities remained in their normal reference ranges. Milk-PL as compared with control fat did not change body weight and BMI during the 8-week intervention. There was a small yet significant decrease in waist circumference.

**Table 4.** Intervention effects of trial 1 (Mean values and standard deviations)

	Control (*n* 31)				Milk-PL (*n* 31)†				
	Day 0		Day 56		Day 0		Day 56		
	Mean	sd	Mean	sd	Mean	sd	Mean	sd	P*
Anthropometric measures and blood pressure									
Body weight (kg)	94·7	12·6	94·5	12·2	94·2	7·4	93·3	7·7	0·181
BMI (kg/m^2^)	30·9	2·9	30·8	2·8	30·7	1·9	30·4	2·0	0·138
Waist circumference (cm)	109·0	8·1	109·1	8·3	110·2	6·1	108·8	6·2	0·001
Body fat mass (%)	30·8	2·4	30·3	2·6	31·2	2·6	30·9	2·8	0·753
Systolic BP (mmHg)	131·7	17·2	131·7	16·4	139·6	21·6	135·3	19·1	0·132
Diastolic BP (mmHg)	82·7	8·9	82·6	8·9	86·4	10·6	84·0	11·0	0·174
Glucose and insulin sensitivity									
Glucose (mmol/l)	5·51	0·47	5·35	0·43	5·49	0·66	5·42	0·63	0·334
Insulin (pmol/l)	104·4	51·3	107·8	98·9	89·2	52·7	92·7	54·4	0·795
HOMA-IR	3·74	2·03	3·74	3·72	3·20	2·06	3·30	2·16	0·495
Plasma lipids and apolipoproteins									
TC (mmol/l)	5·70	0·94	5·72	1·03	6·03	0·82	5·90	1·04	0·302
LDL-C (mmol/l)	3·58	0·76	3·69	0·81	3·78	0·82	3·84	0·89	0·765
HDL-C (mmol/l)	1·32	0·34	1·30	0·28	1·43	0·33	1·42	0·37	0·290
TC:HDL-C ratio	4·50	1·05	4·56	1·20	4·46	1·23	4·40	1·22	0·533
TAG (mmol/l)	1·41	0·49	1·70	1·07	1·46	0·80	1·50	0·64	0·627
PL (mmol/l)	1·88	0·26	1·93	0·37	2·03	0·32	1·97	0·32	0·091
ApoA1 (g/l)	1·56	0·24	1·56	0·22	1·64	0·21	1·61	0·23	0·057
ApoB (g/l)	1·25	0·23	1·26	0·22	1·28	0·27	1·27	0·25	0·571
Biomarkers of inflammation and liver function									
CRP (mg/l)	2·19	2·32	1·42	1·30	2·53	3·03	1·75	1·47	0·947
sICAM (ng/ml)	191·1	31·5	187·6	29·1	189·9	29·3	183·6	25·2	0·426
IL-6 (pg/ml)	1·62	1·48	1·21	0·43	1·34	0·77	1·18	0·54	0·134
Homocysteine (μmol/l)	11·7	3·2	11·8	3·0	11·6	4·0	12·0	3·9	0·547
ALT (U/l)	18·0	8·6	20·9	10·5	17·5	9·9	17·3	7·8	0·057
AST (U/l)	26·2	7·3	25·1	6·2	25·0	7·1	23·5	6·7	0·296
GGT (U/l)	37·1	16·0	43·9	22·8	34·3	15·5	33·2	14·1	<0·001

Milk-PL, milk phospholipids; BP, blood pressure; HOMA-IR, homeostasis model assessment of insulin resistance; TC, total cholesterol; LDL-C, LDL-cholesterol; HDL-C, HDL-cholesterol; PL, phospholipids; CRP, C-reactive protein; sICAM, soluble intracellular adhesion molecule; ALT, alanine transaminase; AST, aspartate transaminase; GGT, γ-glutamyl transferase.

* *P* for intervention effect (time × group). Parameters were not significantly different between the control and milk-PL groups at the start of the trial.

† *n* 30 for IL-6.

### Intervention effects trial 2

Milk-PL as compared with soya-PL did not change the lipid parameters, namely TC, HDL-C, LDL-C, TAG, PL, the primary parameter TC:HDL-C ratio as well as apoA1 and apoB. Furthermore, glucose, insulin and HOMA-IR did not change. Of the inflammation parameters, IL-6 increased in both the milk-PL and soya-PL groups, with no intervention effect. There were no significant changes in CRP, soluble intracellular adhesion molecule, glutathione and tHcy concentrations. Subjects with a mutation in MTHFR 677 (CT and TT) (thirty-four participants) showed an 11 % higher baseline plasma tHcy concentration than those with the wild-type CC (twenty-three participants) (*P* < 0·05). Statistical analysis showed that MTHFR genotype did not modulate the effect of the PL-intervention on plasma tHcy (data not shown). Further, the activity of ALT and AST was also unaffected by intervention. There was a significant intervention effect on GGT activity (*P* < 0·05), in that activity was slightly increased in the soya-PL group and slightly decreased in the milk-PL group (Table 5). No changes were observed in anthropometric measures and blood pressure.

**Table 5.** Intervention effects of trial 2 (Mean values and standard deviations)

	Soya-PL (*n* 29)				Milk-PL (*n* 28)				
	Day 0		Day 49		Day 0		Day 49		
	Mean	sd	Mean	sd	Mean	sd	Mean	sd	P*
Anthropometric measures and blood pressure									
Body weight (kg)	91·0	8·6	91·1	8·9	95·6	10·4	95·4	10·3	0·654
BMI (kg/m^2^)	30·2	2·6	30·0	2·8	30·6	2·3	30·6	2·3	0·444
Waist circumference (cm)	107·3	6·6	107·7	6·8	109·1	6·2	109·4	6·4	0·816
Body fat mass (%)	30·9	2·5	30·5	2·4	31·2	3·3	30·5	2·8	0·395
Systolic BP (mmHg)	144·1	18·6	143·9	16·6	139·6	17·5	140·1	19·8	0·829
Diastolic BP (mmHg)	91·2	8·6	91·3	7·8	91·0	11·7	92·3	12·7	0·346
Glucose and insulin sensitivity									
Glucose (mmol/l)	5·44	0·50	5·33	0·36	5·44	0·59	5·20	0·57	0·254
Insulin (pmol/l)	81·9	33·2	88·8	28·0	80·5	56·4	79·7	42·7	0·609
HOMA-IR	2·89	1·27	2·75	0·97	2·77	1·80	2·65	1·45	0·492
Plasma lipids and apolipoproteins									
TC (mmol/l)	5·46	0·84	5·38	0·77	5·74	0·92	5·65	1·02	0·573
LDL-C (mmol/l)	3·58	0·69	3·65	0·63	3·84	0·76	3·80	0·87	0·372
HDL-C (mmol/l)	1·31	0·32	1·31	0·27	1·32	0·28	1·37	0·28	0·193
TC:HDL-C ratio	4·35	1·04	4·22	0·80	4·46	0·78	4·22	0·83	0·234
TAG (mmol/l)	1·47	0·67	1·55	0·54	1·50	0·57	1·51	0·53	0·632
PL (mmol/l)	2·19	0·36	2·22	0·35	2·24	0·33	2·27	0·41	0·942
ApoA1 (g/l)	1·61	0·22	1·59	0·20	1·63	0·20	1·63	0·22	0·516
ApoB (g/l)	1·33	0·22	1·35	0·20	1·41	0·20	1·38	0·25	0·249
CRP (mg/l)	2·43	2·09	2·80	2·75	2·62	2·59	2·17	1·70	0·576
Biomarkers of inflammation and liver function									
sICAM (ng/ml)	211·2	34·8	205·5	36·7	199·3	37·7	197·3	35·1	0·449
IL-6 (pg/ml)	1·58	0·86	1·74	1·16	1·33	0·77	1·45	0·65	0·151
Homocysteine (μmol/l)	13·9	5·0	13·7	4·7	12·5	4·0	12·4	3·5	0·962
Glutathione (mmol/l WB)	0·67	0·13	0·69	0·14	0·70	0·15	0·71	0·16	0·714
ALT (U/l)	13·9	5·2	15·9	6·9	12·0	4·9	13·2	6·6	0·615
AST (U/l)	25·3	5·5	26·1	5·6	24·9	6·4	25·3	7·9	0·442
GGT (U/l)	36·5	15·6	37·9	16·9	33·4	15·4	32·0	13·0	0·042

Soya-PL, soyabean phospholipids; milk-PL, milk phospholipids; BP, blood pressure; HOMA-IR, homeostasis model assessment of insulin resistance; TC, total cholesterol; LDL-C, LDL-cholesterol; HDL-C, HDL-cholesterol; PL, phospholipids; CRP, C-reactive protein; sICAM, soluble intracellular adhesion molecule; WB, whole blood; ALT, alanine transaminase; AST, aspartate transaminase; GGT, γ-glutamyl transferase.

* *P* for intervention effect (time × group). Parameters were not significantly different between the soya-PL and the milk-PL groups at the start of the trial.

## Discussion

Milk-PL and soya-PL intakes did not change lipid and apolipoprotein concentrations in our trials. In other well-designed human studies the effect of milk-PL was also moderate or non-existent. The consumption of a drink with 2·8 g/d of milk-PL (rich in SM) as compared with a control drink containing the same amount of PL low in SM (66 % egg-PL) for 3 weeks did not significantly change the concentrations of TC, LDL-C, HDL-C, TAG, apoA1 and apoB^(^^14^^)^. In subjects with atopic dermatitis, 3 g/d milk-PL did not improve TC, LDL-C, HDL-C or TAG^(^^13^^)^. In a short pilot study, 1 g/d of milk-SM increased HDL-C by approximately 13 %, but did not change the TC:HDL-C ratio^(^^38^^)^. However, in participants who consumed a drink providing either 45 g/d of MFGM-rich buttermilk solids (188 mg/d PL) or 45 g/d of a macro-/micronutrient-matched dairy placebo for 4 weeks, the buttermilk as compared with the control drink reduced TC and LDL-C by 3·1 %, and TAG by 10·7 %^(^^15^^)^. In another study the participants were randomised to one of three dietary regimens, either to no change in the dietary habits (control) or one extra egg per d or 100 ml buttermilk (approximately 90 mg PL per d) together with one egg yolk per d for 12 weeks. In female subjects the regular egg consumption increased TC and LDL-C as compared with the control group, while no such increase was observed in the buttermilk–egg yolk group. Similarly, milk fat with intact MFGM structures (41·8 g fat/d, 19·8 mg PL/d) did not increase TC and LDL-C in human subjects, while the same amount of pure butter oil did so. Furthermore, suppression of a number of genes in peripheral blood mononuclear cells was correlated with changes in lipids^(^^17^^)^.

Our subjects were somewhat older and more overweight than those of other studies examining the effect of milk-PL^(^^13^^–^^17^^)^ or pure milk-SM^(^^38^^)^, had higher plasma TAG concentrations and somewhat or clearly higher TC:HDL-C ratios. Baumgartner *et al*.^(^^16^^)^ observed a protective effect of milk-PL only in females, but other studies using milk-PL^(^^14^^,^^15^^)^ or milk-SM^(^^38^^)^, as well as studies using soya-PL^(^^6^^,^^7^^,^^39^^)^ gave little indication that males and females respond differently.

Our trials, like two others^(^^13^^,^^14^^)^, used milk-PL concentrates isolated from butter serum as test material, while again others used buttermilk^(^^15^^,^^16^^)^ or cream with MFGM^(^^17^^)^. An *in vitro* precursor study suggested that extensive processing may impair the cholesterol-lowering activity of MFGM from buttermilk, possibly through proteins adsorbing at the surface of MFGM fragments^(^^40^^)^. Concentrated isolated MFGM material may thus have differing structural properties than liquid forms in buttermilk or cream, which may in turn affect lipid metabolism differently^(^^41^^)^. It is a tempting speculation that this difference in the food matrix may have contributed to the lack of effect in our study and others^(^^13^^,^^14^^)^. But there is no basis for such an assumption without further appropriate experiments. An alternative explanation would be that other components than PL in buttermilk may contribute to the effects. Buttermilk is usually fermented and contains lactic acid bacteria. Some of them were reported to affect lipid metabolism^(^^42^^)^.

In trial 1 milk-PL reduced waist circumference slightly yet significantly. However, there was no such effect compared with soya-PL in trial 2. Blood pressure did not change in either trial. In the study of Conway *et al*.^(^^43^^)^ a buttermilk supplement reduced systolic blood pressure and also decreased concentration of the angiotensin I-converting enzyme in normotensive subjects. The authors attributed these effects to the proteins of MFGM, as there is evidence that milk peptides can reduce blood pressure (for a review, see Conway *et al*.^(^^43^^)^). Beneficial effects of dairy product intake on body weight and body composition are often attributed to the Ca content^(^^44^^)^, but also to proteins in milk and milk products^(^^45^^)^.

The only parameters of liver function determined in our study were liver enzyme activities in serum. Elevated liver enzyme activities, in particular ALT and GGT, are indicators of fatty liver. We observed a reduced GGT activity in the milk-PL group as compared with the control group in trial 1, and there was also a reduced activity in the milk-PL group as compared with the soya-PL group in trial 2. Yet all enzyme activities remained within the normal physiological range, and the fatty liver index did not change. ALT, AST or GGT activities were not modified by buttermilk PL in one study^(^^16^^)^, or were not examined at all in other studies^(^^13^^–^^15^^,^^43^^)^.

As mentioned before, PL and SM are a source of choline, and may affect liver function also via their role in choline metabolism. As little as 300 mg supplemental choline/d was sufficient to avoid increased serum ALT, AST or GGT activities^(^^28^^)^. The majority of our subjects consumed more than this amount. Rat or mouse experiments, which demonstrated decreased AST and ALT activities^(^^19^^)^ or decreased liver lipids following a diet with supplemental soya-PL^(^^19^^)^, milk-PL^(^^11^^,^^12^^,^^20^^)^ or SM^(^^21^^)^, were performed in animals with enhanced susceptibility due to either genetic disposition^(^^12^^,^^21^^)^ or diets high in ethanol or fat^(^^11^^,^^19^^,^^20^^)^. Milk-PL supplements had no effects on liver lipids in rats on a normal chow diet^(^^11^^)^.

Choline plays a pivotal role as a methyl donor for the remethylation of Hcy to methionine. Moderately elevated concentrations of plasma tHcy have been linked to a variety of disease conditions, including CVD. Higher choline plus betaine intakes (within the normal range) were associated with moderately lower plasma tHcy concentration as well as lower concentrations of inflammatory markers like IL-6 and TNF-α in an observational study^(^^46^^)^. A high choline supplement attenuated the post-methionine load-induced increase in plasma tHcy^(^^28^^)^, while a choline-deficient diet increased plasma tHcy^(^^25^^)^. Participants in our trials had a choline intake comparable with or even higher than the cohort of Detopoulou *et al*.^(^^46^^)^, but intake of PL and choline was highly variable between participants. Considering that also individual requirements are variable^(^^26^^)^, it is not surprising that the additional choline provided by the soya-PL or milk-PL supplements did not modify the concentration of tHcy or inflammatory parameters.

MTHFR 677 genotype may modify plasma tHcy. Baseline plasma tHcy concentrations were modestly higher in men with the MTHFR 677TT relative to men with the 677CC genotype, and increased more strongly in response to low folate intake^(^^27^^)^. A post-methionine load-induced increase of plasma tHcy was less pronounced in men with the MTHFR 677TT than in those with the 677CC genotype^(^^28^^)^. We observed only small yet significantly higher baseline plasma tHcy concentrations in men with the MTHFR 677CT than in those with the 677CC genotype in trial 2. Yet the group size for genotyping was small. Furthermore, in this study almost only 677CT heterozygotes rather than 677TT homozygotes were compared with 677CC homozygotes. Genetic analyses took place after the study had been performed and with knowledge about the general results of the analyses. Anyhow, the genotype did not modulate any intervention effect. Strengths of this study are the well-characterised test material with a closely matched control, the fairly long intervention periods, and the well-documented dietary habits. Potential limitations are variability of dietary habits and thus background diet and further lifestyle factors, including physical activity. Furthermore, only middle-aged overweight male volunteers were included in the studies. In conclusion, under these conditions dietary supplementation with milk-PL as compared with control milk fat reduced waist circumference and, as compared with both control fat and soya-PL, GGT activity.
